# Supplementary figures and images for: An oral cholera vaccine in the prevention and/or treatment of inflammatory bowel disease
Source: PLoS One. 2023 Aug 28;18(8):e0283489. doi: 10.1371/journal.pone.0283489 (PMC10461820; doi:10.1371/journal.pone.0283489)

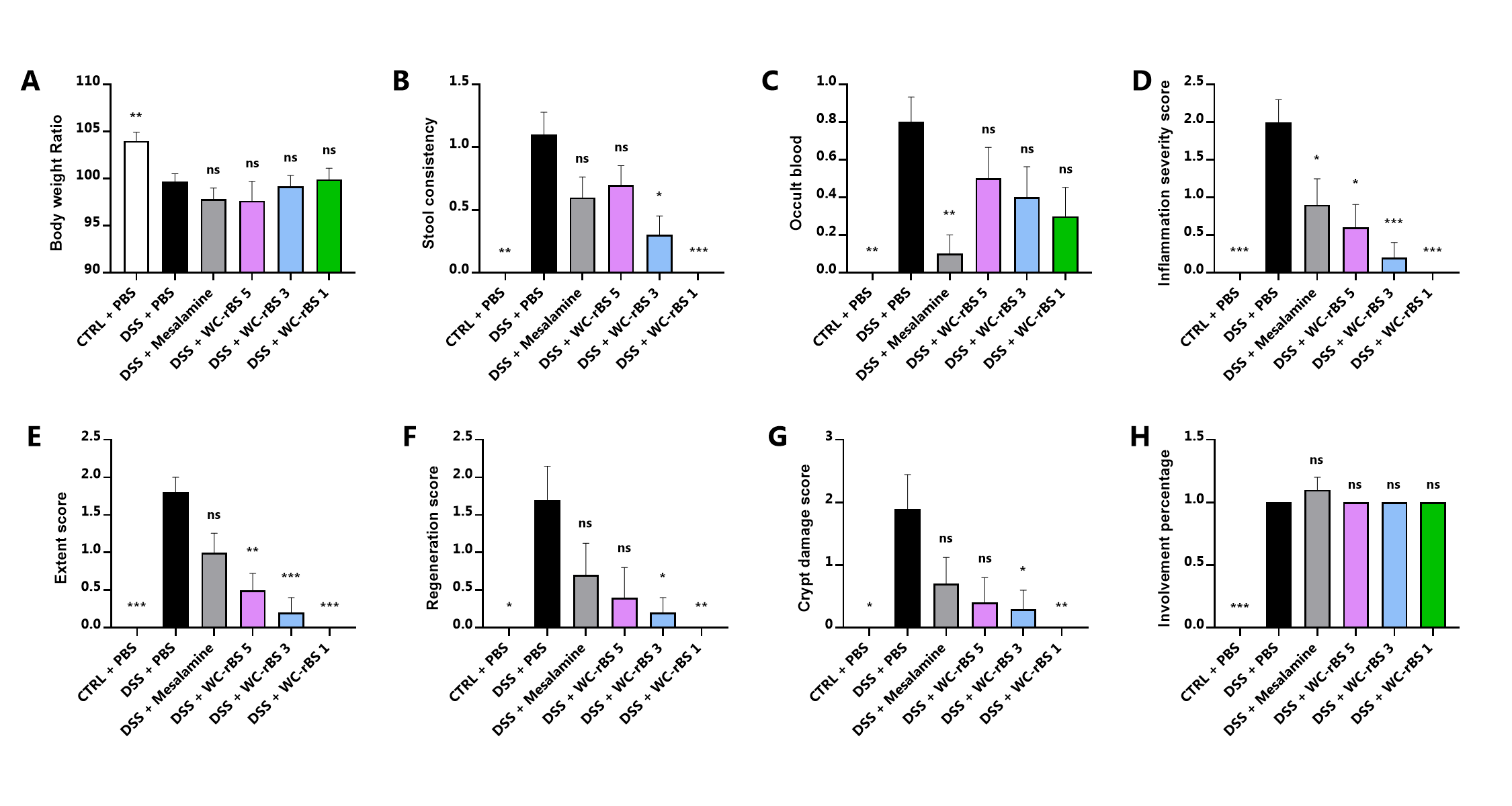

Supplement: S1 Fig — Day 12 DAI and histological scores reported in Fig 3 are calculated as the sum of different parameters. DAI is the sum of the following parameters: the body weight ratio between day 12 and day 0: the first day of DSS administration (A), the stool consistency (B) and the presence of occult blood (C). The global histological score refers to the sum of the severity (D) and extent (E) of inflammation, the epithelium regeneration (F), the crypt damages (G) and the percentage of involvement (H). Bars represent mean ±SEM. Statistics: Permutation test. ns: non-significant, * p-value < 0.05, ** p-value < 0.01, *** p-value < 0.001. (TIFF) [file pone.0283489.s001.tiff]

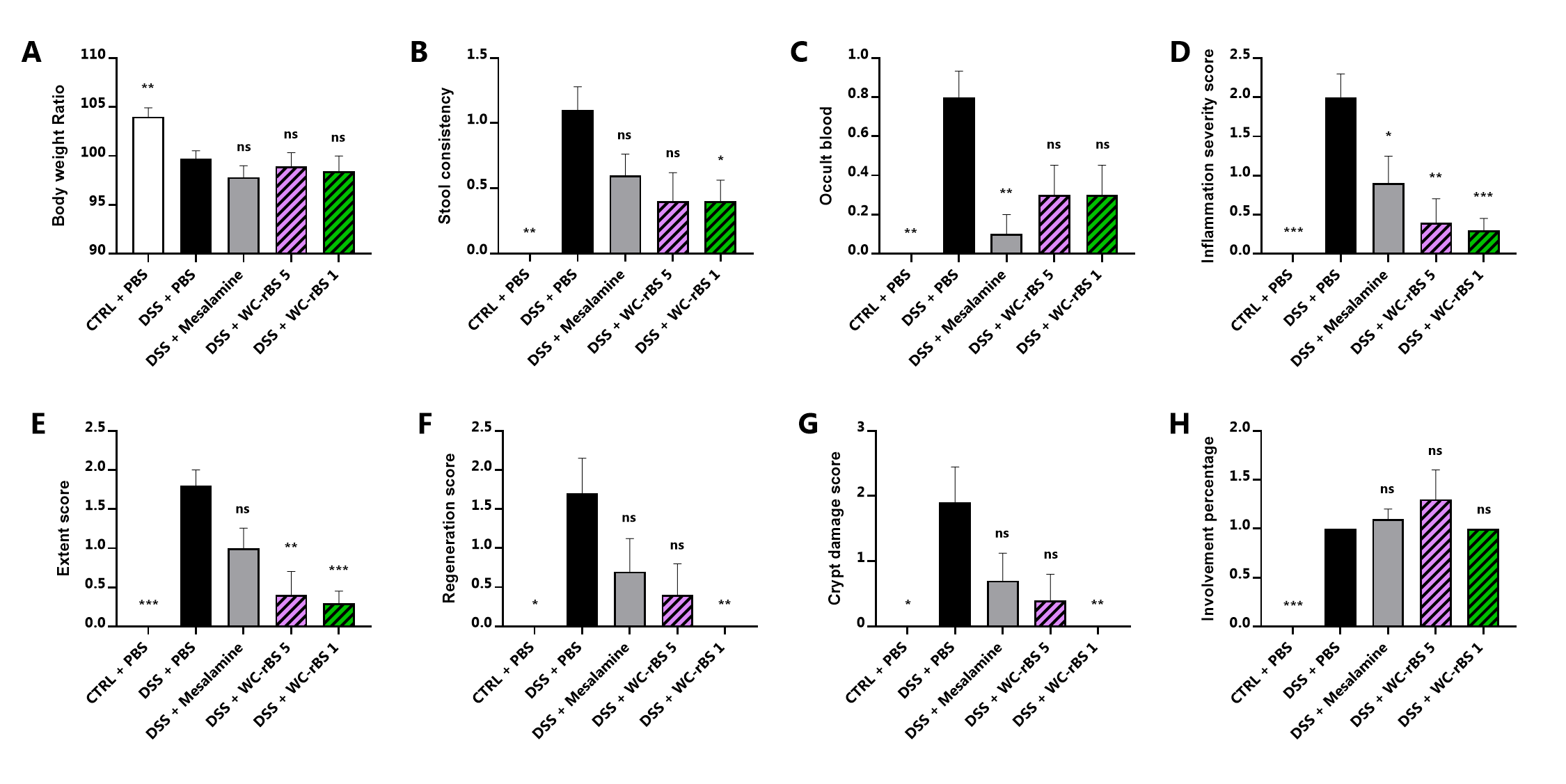

Supplement: S2 Fig — Day 12 DAI and histological scores reported in Fig 4 are calculated as the sum of different parameters. DAI is the sum of the following parameters: the body weight ratio between day 12 and day 0: the first day of DSS administration (A), the stool consistency (B) and the presence of occult blood (C). The global histological score refers to the sum of the severity (D) and extent (E) of inflammation, the epithelium regeneration (F), the crypt damages (G) and the percentage of involvement (H). Bars represent mean ±SEM. Statistics: Permutation test. ns: non-significant, * p-value < 0.05, ** p-value < 0.01, *** p-value < 0.001. (TIFF) [file pone.0283489.s002.tiff]

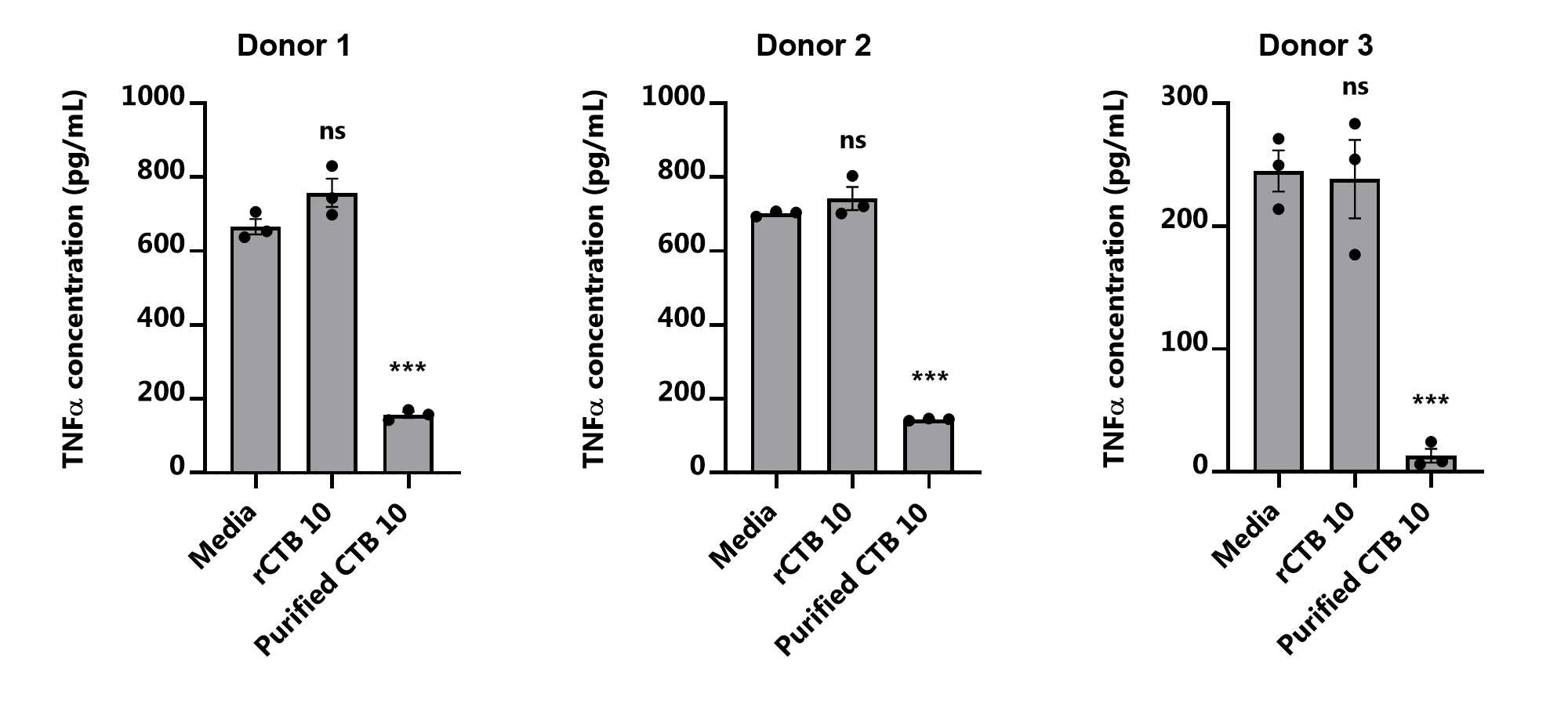

Supplement: S3 Fig — PBMCs from 3 different healthy donors were pretreated for 16h with media, rCTB (10μg/mL), or purified CTB from CT (Sigma C9903, 10μg/mL) and then challenged with LPS (1μg/mL). Supernatants were collected 6h post challenge to assess TNFα concentration. Bars represent mean ±SEM. Statistics: One-way ANOVA with Dunnett’s multiple comparison. ns: non-significant, *** p-value < 0.001. (TIFF) [file pone.0283489.s003.tiff]
