# Supplementary material for: An oral cholera vaccine in the prevention and/or treatment of inflammatory bowel disease
Source: PLoS One. 2023 Aug 28;18(8):e0283489. doi: 10.1371/journal.pone.0283489 (PMC10461820; doi:10.1371/journal.pone.0283489)
Supplement: Attachment — Submitted filename: PONE-D-23-07112.docx [file pone.0283489.s005.docx]

PONE-D-23-07112

The manuscript entitled " An oral cholera vaccine in the prevention and/or treatment of inflammatory bowel disease" by Meunier et al. describes studies aimed to investigate the potential beneficial 101 properties of WC-rBS vaccine in autoimmune disease. In general, the subject is interesting and it is the first report of the therapeutic effect of WC-rBS – a prophylactic oral cholera vaccine – for the treatment of intestinal colitis. This paper is clear and straightforward, the parameters asserted are clear and the data well represented. However, there are some problems in this study should be considered before this manuscript being accepted*.*

Comments:

Page 9 L49: E. coli should be in italic … You should review all bacterial names.

Page 10 L79: Before start use the abbreviation of *V. cholerae*, you should mention before the use of this abbreviation “*Vibrio cholerae* (*V. cholerae)”.*

Page 11 L91: The author should correct: TGF-β.

Page 11 L108: “To our knowledge, this is the first report of the therapeutic effect of WC-rBS – a prophylactic oral cholera vaccine – for the treatment of intestinal colitis” … Better to say for the treatment and prevention of intestinal colitis.

Page 11: Add a reference for the protocol of isolation and use of PBMC

Figure 3: Please add the protocol like Figure 2 and 3

In the PBMC and TPH-1 *in vitro* experiment, I recommend to test another cytokine mainly IL-12, INF-γ and calculate the IL-10/IL-12 ratio.

In the DSS *in vivo* experiment, auteur should add a representative histological section for each group and it is very important to add the Shortening of colon length if available and test different genes expression like CXCL2, IL-6 for inflammation and ZO-1, occludine from colonic samples.

In the DSS *in vivo* experiment, why you orally administrate WC-rBS on days -6, -3, 0, 3 and 6 not every day? And why the mice were exposed to 2.5% DSS not for example 3.5% or 5%?

Why the auteur uses DSS colitis mouse model not TNBS colitis mouse model?
